# Premenstrual dysphoric disorder is associated with the longer length from clitoris to urethra

**DOI:** 10.1186/s12905-021-01403-4

**Published:** 2021-07-05

**Authors:** Zheng Li, Meng-jiao Xu, Ying Jin, Bing-gen Zhu

**Affiliations:** 1 Pudong New Area Mental Health Center, Tongji University School of Medicine, Shanghai, 200124 China; 2 Department of Gynaecology and Obstetrics, Tongji Hospital, Tongji University School of Medicine, Shanghai, 200065 China; 3 Department of Psychiatry, Tenth Peoples’ Hospital, Tongji University School of Medicine, Shanghai, 200072 China

**Keywords:** Premenstrual dysphoric disorder, Length from clitoris to urethra, Anogenital distance, 2D:4D ratios

## Abstract

**Background:**

Premenstrual dysphoric disorder (PMDD) is a common, recently recognized, psychiatric condition among reproductive women, reflecting abnormal responsivity to ovarian steroids. Moreover, the potential organizational effect of prenatal sex hormones during PMDD has got attentions, but there have been considerably less of researches on this topic. The aim of this research was to investigate the possible role of prenatal androgen in the PMDD.

**Methods:**

Anogenital distance (AGD), the distance between a woman’s clitoris and her urethral meatus (CUMD), left and right 2D:4D ratios were measured in 77 subjects (25 patients with PMDD), as these anthropometric indicators are considered to indirectly reflect prenatal androgen exposures in utero.

**Results:**

Patients with PMDD had a longer CUMD than controls (25.03 ± 4.73 vs. 22.07 ± 4.30, *P* = 0.008), while there were no significant difference between PMDD group and control group in the AGD and right and left 2D:4D ratios.

**Conclusion:**

Atypical high prenatal androgen exposure might predispose individuals to be susceptible to PMDD.

**Supplementary Information:**

The online version contains supplementary material available at 10.1186/s12905-021-01403-4.

## Background

Premenstrual dysphoric disorder (PMDD) is a severe form of premenstrual syndrome, characterized by mood symptoms appearing in a cyclic manner during the premenstrual period [1]. The irritability and anger are regarded as the cardinal symptoms, and depressed mood, tension and affect lability are also common complaints [1, 2]. 3–5% of women of menstrual age may suffer from the disorder. These symptoms of PMDD significantly impair daily functioning, including the sexual dissatisfaction [1, 2]. PMDD has been recently designated as a separate entity under Depressive disorders in the Diagnostic and Statistical Manual of Mental Disorders, Fifth Edition (DSM-5) (http://www.dsm5.org/Pages/Default.aspx) [3]. The etiology of PMDD is unclear. Potential biological contributors include central nervous system sensitivity to reproductive hormones, genetic factors, and psychosocial factors such as stress [4].

It is well-known that the actions of the prenatal hormones are organizational and enduring [5]. Several studies have implicated that the organizational effects of prenatal sex hormones might be one of predisposing factors that underlie the behavioral changes in reproductive-age women due to fluctuation of sex hormones [6–8]. Kaneoke et al measured the second to fourth digit ratios (2D:4D) to investigate the role of prenatal sex hormone in the pathogenesis of PMDD [8], as a number of studies have supported that 2D:4D is a biomarker for the balance between fetal testosterone and estrogen. They found that right- and left-hand 2D:4D were differentially related to the severity of premenstrual symptoms, and the prenatal sex hormones (e.g., testosterone and estrogen) exposure might contribute to individual differences in the severity of premenstrual symptoms. A related neuroimaging study revealed that the network properties of the temporal region in the females with high 2D:4D digit ratio was significantly different from the low 2D:4D digit ratio ones [9]. Naturally occurring and experimentally induced rhesus macaque models for polycystic ovary syndrome (PCOS) testified that higher testosterone at early-to-med gestation was not only involved in PCOS-like symptoms, but also related to behavioral changes (such as depression) and elongated anogeniatl distances exhibited by the adult female rhesus macaque [10]. These researches suggested that prenatal sex hormone exposure deserved attentions for investigating the mechanisms underlying the variable prevalence and symptoms of gynecological and psychiatric diseases.

Anogenital distance (AGD) is a sexually dimorphic phenotype with males’ AGD measuring longer than females, and considered as a sensitive marker of in utero exposure to androgens, based on animal models and the human literature [11, 12]. The distance between a woman’s clitoris and her urethral meatus (CUMD) is a segment of AGD, and has been also considered to likely reflect the extent of prenatal androgen exposure [13]. Based on many recent researches, the AGD rather than the 2:4 digit ratios is more likely to provide an accurate biomarker of fetal androgen exposure in humans [11, 14, 15]. To our knowledge, no published work has examined the association between the CUMD/AGD and PMDD.

## Methods

### Participants and grouping

The 77 participants are women who visited the gynecological or psychosomatic clinic of hospitals between June 2018 and June 2020 and agreed on participation after listening to explanations about the study. Eligibility criteria included age 18–45, regularly menstruating, nulliparous with no pregnancy lasting more than 10 weeks, not currently receiving any treatments to control the secretion of hormones (including taking birth control pills or an injectable contraception, administering gonadotropin releasing hormone, hormone replacement therapy, etc.), no evidence of any hormonal disorder (including PCOS), no history of injury to or surgery on the genital region, no history of congenital anatomical abnormalities in genital organs including Mullerine Agenesis, and no history of an injury to the 2nd or 4th digit of either hands. These inclusion criteria for subjects referred to the literature [16], was used in our other related studies (unpublished work; https://www.researchsquare.com/article/rs-230184/v1).

25 participants had symptoms in the past year and next two menstrual cycles, that corresponded to diagnosis criteria of PMDD, while other 52 participants were regarded as control group, as they did not present any premenstrual symptoms, or their manifestations were insufficient to diagnostic standards of PMDD. The diagnosis of PMDD is based on the fulfillment of seven (A to G) criteria (seen in the Additional file 1), as described in the DSM-5 (http://www.dsm5.org/Pages/Default.aspx) [3].

### Anthropometry

**Fig. 1:**
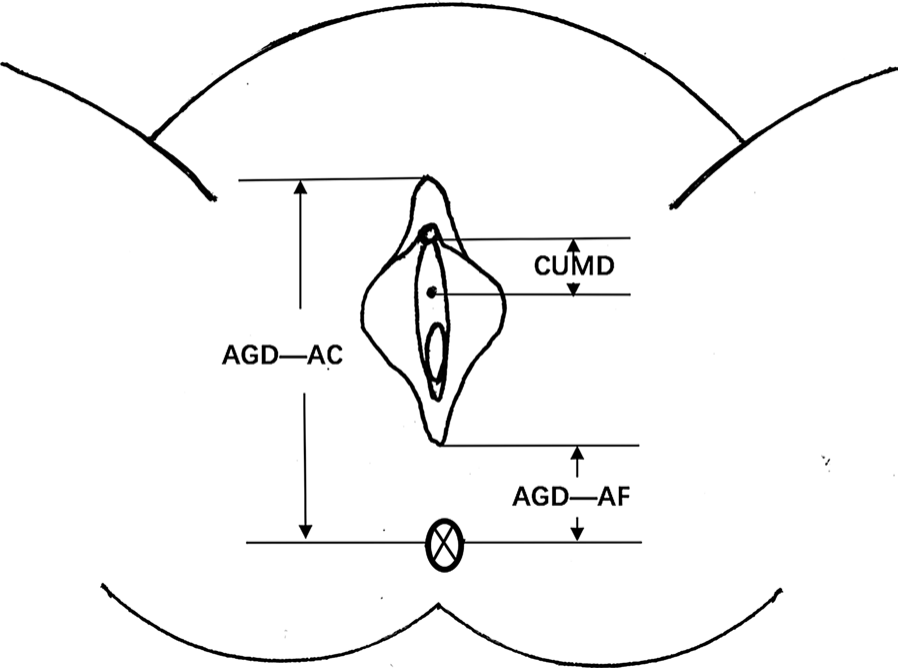
The graphic displayed measurements of CUMD, AGD-AC and AGD-AF. CUMD, from the underside of clitoral glans to the center of the urinary meatus; AGD-AC, from the center of the anus to the anterior clitoral surface; AGD-AF, from the center of the anus to the posterior fourchette

Anthropometric data was collected at study enrollment. The AGD and CUMD measurements were taken using a digital caliper (Carbon Fiber Composites Digital Caliper, Wuxi Kaibaoding Tool Limited Company, Jiangsu, China) in millimeter (mm), following procedures described elsewhere [13, 16]. The subjects were first placed in the supine position and changed to the lithotomy position in which the legs are spread apart to be put on rests. The AGD-AC was measured as the distance from the center of the anus to the anterior clitoral surface (Fig. 1); The AGD-AF was measured as the distance from the center of the anus to the posterior fourchette. The CUMD was measured as the distance from the underside of clitoral glans to the center of the urinary meatus. In order to improve accuracy, measurements were collected in triplicate by two independent examiners, the mean value of the six measurements of each distance was used.

Measurement of Digit Ratio: A photograph of both hands was taken using a digital camera. The hands were held in supination and fingers completely extended. The lengths of index and ring fingers of both hands were measured from the bottom crease of each digit to the finger tip, using tools in Adobe Photoshop [17]. For each hand, the digit ratio (2D:4D) was subsequently calculated, namely dividing index finger (2D) length by ring finger (4D) length. Mean of right and left hand ratio was taken as mean 2D:4D ratio for each individual.

These anthropometric methods, including measurements of CUMD, AGD-AC/AGD-AF, and Digit Ratio referred to the previous researches [13, 16, 17], were also used in our other related studies (unpublished work; https://www.researchsquare.com/article/rs-230184/v1).

### Statistical analyses

All analyses were conducted in SPSS Version 23. The variables were summarized by arithmetic mean ± standard deviation (SD). Unpaired Student's *t*-tests were used for comparison of variables between cases and controls. A P value of < 0.05 was deemed significant.

## Results

The general characteristics of controls and cases with PMDD were shown in Table 1. The demographic data, including ages, menarche age, height, weight and BMI in the PMDD group were not significant different from that in the control group. Patients with PMDD had a longer CUMD than controls (25.03 ± 4.73 vs. 22.07 ± 4.30, *P* = 0.008), while there were no significant difference between PMDD group and control group, as to AGD-AC, AGD-AF, right and left 2D:4D ratio.

**Table 1:** Comparison of the general characteristics of controls and cases with premenstrual dysphoric disorder (PMDD).

Variable	PMDD group, *n = 25*	Control group, *n = 52*	*P* value
Age (years)	27.88 ± 6.90	29.52 ± 6.56	0.316
Menarche Age (years)	13.21 ± 1.06	13.17 ± 1.17	0.900
Height (cm)	162.80 ± 5.34	162.73 ± 5.66	0.959
Weight (kg)	58.10 ± 8.45	57.87 ± 10.83	0.924
BMI (kg/m^2^)	21.92 ± 2.93	21.86 ± 4.03	0.951
CUMD (mm)	25.03 ± 4.73	22.07 ± 4.30	0.008
AGD-AC (mm)	94.55 ± 11.25	94.86 ± 10.54	0.906
AGD-AF (mm)	28.62 ± 6.57	30.07 ± 5.76	0.327
Left2D:4D ratio	0.976 ± 0.037	0.980 ± 0.044	0.667
Right2D:4D ratio	0.972 ± 0.032	0.967 ± 0.037	0.516

## Discussion

The PMDD is triggered at times of hormonal fluctuations, in particular exposure to progesterone [1], and does not occur in the women during pregnancy and after menopause (without hormone replacement). The suppression of ovulation and suppression of the cyclical hormonal changes by the hormone therapy is the most effective treatment for PMDD [1, 18]. These evidences highlight that the direct activating effect of hormones plays an essential role in the pathogenesis of PMDD, at least sexual steroid hormones, such as progesterone, may precipitate the presentation of PMDD. Moreover, the very early organizational effect of prenatal sex hormones that may contribute to the PMDD has recently got attentions. The previous work using the 2D:4D ratios as a marker of prenatal sex hormones exposure, indicated that the prenatal sex hormones exposure might influence individual differences in the severity of premenstrual symptoms [9], and could be a factor in the development of PMDD. A recent preclinical animal study clearly showed that prenatal androgenization induced anxiety-like behavior in adult female rats, implying that prenatal exposure to high concentration of testosterone might influence the development of neural networks and impose the risk of anxiety-like behavior later in life [19]. We measured the 2D:4D ratios, AGD-AC, AGD-AF, and CUMD of the subjects, and found that the left/right 2D:4D ratios, AGD-AC and AGD-AF did not show any difference between PMDD patients and controls, but a significant longer CUMD was seen in the patients with PMDD. The CUMD, as well as the 2D:4D ratios, AGD-AC and AGD-AF, is supposed to be positive association with prenatal androgen levels, therefore, our results supported that atypical high prenatal androgen exposure might predispose individuals to be susceptible to PMDD.

We did not detect the current level of androgens in the patients with PMDD. An early research reported that serum levels of androgens were higher in women with premenstrual irritability and dysphoria than in controls [20], but other studies had shown that plasma testosterone in women with premenstrual symptoms was not different from that in non-symptomatic controls [21, 22]. A recent study which carefully investigated the level of androgens in women with cyclical mood changes and premenstrual syndrome demonstrated that plasma testosterone was significantly lower in women with luteal phase symptoms compared with those with additional follicular phase symptoms [23]. The PCOS is a hyperandrogenic, oligomenorrhea/amenorrhea, fertility problems and metabolic disorder found in 6–7% of reproductive-aged women [24]. Therefore, the clinical features and pathophysiological processes of the PMDD should be totally different from ones of the PCOS. Recently, several studies demonstrated that AGD in adult patients with PCOS was longer than that in control, implying that extreme prenatal androgen exposure might contribute to PCOS [16, 25, 26]. Whereas, our PMDD patients showed elongated CUMD, rather than extended AGD. It seems that both of PMDD and PCOS are probably involved in the higher prenatal androgen exposures. But, why did PMDD patients have a longer CUMD and PCOS patents have a longer AGD? Future studies are awaited to help delineate the difference. At present, it can be inferred that there are other factors led to discrepant perineum appearances, in addition to prenatal androgen hormones.

Several reports demonstrated that the presence of premenstrual symptoms correlated negatively with sexual satisfaction [23, 27–29]. Sexual pleasure and orgasm during copulation in women depends on many factors, such as past experience, stimulation of one or all of these triggering zones, autonomic arousal, and partner- and contextual-related cues, etc. [30]. The clitoral complex in relation to the urethra, vulva, and vagina is the essential sensory triggering zone [29]. A longer CUMD in a woman decrease her likelihood of experiencing orgasm in sexual intercourse, as the longer CUMD may decrease penile-clitoral contact during sexual intercourse or decrease penile stimulation of internal aspects of the clitoris [12]. Therefore, the longer CUMD might contribute to the sexual difficulties of women with premenstrual symptoms, according to our results.

There are some major defects in the present studies. This is a preliminary study with limited samples that were not chosen randomly, the way of collecting cases could lead the bias to some extent. Moreover, we did not study the association of individual differences in the severity of premenstrual symptoms with the left/right 2D:4D ratios, AGD-AC, AGD-AF and CUMD, and did not collect data about the sexual function/satisfaction of subjects, at same time.

## Conclusion

The PMDD was associated with the longer CUMD, it might be one of underling factors which induced sexual difficulties of women with PMDD. More importantly, our results supported that atypical high prenatal androgen exposure might predispose individuals to be susceptible to PMDD.

## Supplementary Information

**Additional file 1: **Diagnostic criteria for PMDD.  

## Data Availability

The data and materials described in the current study are available from the corresponding author on reasonable request.

## Abbreviations

AGD: Anogenital distance
AGD-AC: Distance from the center of the anus to the anterior clitoral surface
AGD-AF: Distance from the center of the anus to the posterior fourchette
BMI: Body mass index
CUMD: Distance between a woman’s clitoris and her urethral meatus
DSM-5: Depressive disorders in the Diagnostic and Statistical Manual of Mental Disorders, Fifth Edition
PCOS: Polycystic ovarian syndrome
PMDD: Premenstrual dysphoric disorder
2D:4D: Second to fourth digit ratios
