# Supplementary material for: Premenstrual dysphoric disorder is associated with the longer length from clitoris to urethra
Source: BMC Womens Health. 2021 Jul 5;21:266. doi: 10.1186/s12905-021-01403-4 (PMC8259395; doi:10.1186/s12905-021-01403-4)
Supplement: Supplementary file 1 — Additional file 1: Diagnostic criteria for PMDD. [file 12905_2021_1403_MOESM1_ESM.doc]

The supplement file: Diagnostic Criteria for PMDD

| Items | Requirements | Symptoms |
| --- | --- | --- |
| Criterion A | At least five symptoms in most menstrual cycles and to stage-specificity of the cycle in the past year | Marked lability (e.g., mood swings).  Marked irritability or anger.  Markedly depressed mood.  Marked anxiety and tension.  Decreased interest in usual activities.  Difficulty in concentration.  Lethargy and marked lack of energy.  Marked change in appetite (e.g., overeating or specific food cravings).  Hypersomnia or insomnia.  Feeling overwhelmed or out of control.  Physical symptoms (e.g., breast tenderness or swelling, joint or muscle pain, a sensation of ‘bloating’ and weight gain). |
| Criterion B | One (or more) specific symptoms of the disorder, must be present | Marked affective lability.  Marked irritability or anger or increased interpersonal conflicts.  Marked depressed mood, feelings of hopelessness, or self-deprecating thoughts.  Marked anxiety, tension, and/or feelings of being keyed up or on edge. |
| Criterion C | One (or more) symptoms must be additionally be present, to reach a total of *five* symptoms when combined with symptoms from Criterion B above. | Decreased interest in usual activities (e.g., work, school, friends, hobbies).  Subjective difficulty in concentration.  Lethargy, easy fatigability, or marked lack of energy.  Marked change in appetite; overeating; or specific food cravings.  Hypersomnia or insomnia.  A sense of being overwhelmed or out of control.  Physical symptoms such as breast tenderness or swelling, joint or muscle pain, a sensation of "bloating," or weight gain. |
| Criterion D | Underscores the clinical significance or interference of symptoms with daily-life activities (e.g., avoidance of social activities; decreased productivity and efficiency at work, school, or home). |  |
| Criterion E | Confirm the specificity of PMDD as compared with mood and anxiety disorders, etc. |  |
| Criterion F | Requests the existence of two month's daily prospective ratings |  |
| Criterion G | The absence of a medical or drug-induced cause of the disorder |  |
